# Trial of Subcutaneous Ketamine for Psychotic Depression in an Older Adult With Comorbid Physical Illness: A Case Report

**DOI:** 10.1192/bjo.2026.11780

**Published:** 2026-06-30

**Authors:** Roshan Ganeshan, Tharun Zacharia

**Affiliations:** Older Adults Mental Health Liaison, Guys and St Thomas NHS Foundation Trust, London, United Kingdom

## Abstract

**Aims:**

Ketamine is an N-methyl-D-aspartate (NMDA) receptor antagonist used for anaesthesia and analgesia, with increasing interest in its rapid antidepressant effects. This case evaluates the feasibility, tolerability and clinical response to subcutaneous ketamine in a medically frail older adult with psychotic depression, hypothesising that ketamine would rapidly improve engagement and depressive burden to support nutritional intake and medical recovery.

**Methods:**

A 77-year-old man with a prior severe episode of psychotic depression had previously achieved full recovery after eight sessions of electroconvulsive therapy (ECT). In the current episode, he presented similarly with disorientation, nihilistic beliefs, poor engagement, self-neglect and severe weight loss from reduced oral intake, consistent with a depressive relapse with psychotic features.

His case was complicated by medical instability with suspected delirium, as well as limited adherence to and response from conventional antidepressant treatment. Anaesthetic risk in the context of acute physical illness, led to ECT being deferred and subcutaneous ketamine being used as a practical ward-based intervention when urgent treatment was required.

Following multidisciplinary and family discussion, subcutaneous ketamine was initiated as a rapid antidepressant intervention. Three doses were administered over approximately ten days (two at 0.25 mg/kg, followed by escalation to 0.5 mg/kg due to limited initial response). Depressive symptoms were rated using the Cornell Scale for Depression in Dementia (CSDD), alongside serial clinical assessments of mental state, engagement and oral intake.

**Results:**

Treatment was well tolerated, with no dissociative or behavioural adverse effects observed. CSDD scores showed modest change, decreasing from 15 at baseline to 12 after the first dose and to 10 after the third and final dose, with transient improvements in engagement and acceptance of care that were not sustained and did not translate into lasting improvement in overall mental state or nutritional intake.

**Conclusion:**

Subcutaneous ketamine was a feasible and well-tolerated option in this medically frail older adult but did not achieve a clear or sustained antidepressant response in this case. Given the patient’s prior robust response to ECT and the limited benefits observed with ketamine, referral for ECT was re-initiated once anaesthetic risk was acceptable, highlighting the potential role of subcutaneous ketamine as a short-term bridging intervention when ECT is temporarily high-risk and the need for further evidence in older adults with psychotic depression and comorbid physical illness.

